# Supplementary material for: Fetal Biometric Assessment and Infant Developmental Prognosis of the Tadalafil Treatment for Fetal Growth Restriction
Source: Medicina (Kaunas). 2023 May 8;59(5):900. doi: 10.3390/medicina59050900 (PMC10223650; doi:10.3390/medicina59050900)
Supplement: Supplementary file 1 [file medicina-59-00900-s001.zip › Supplemental_Appendix_Figure.pptx]

## Slide 1
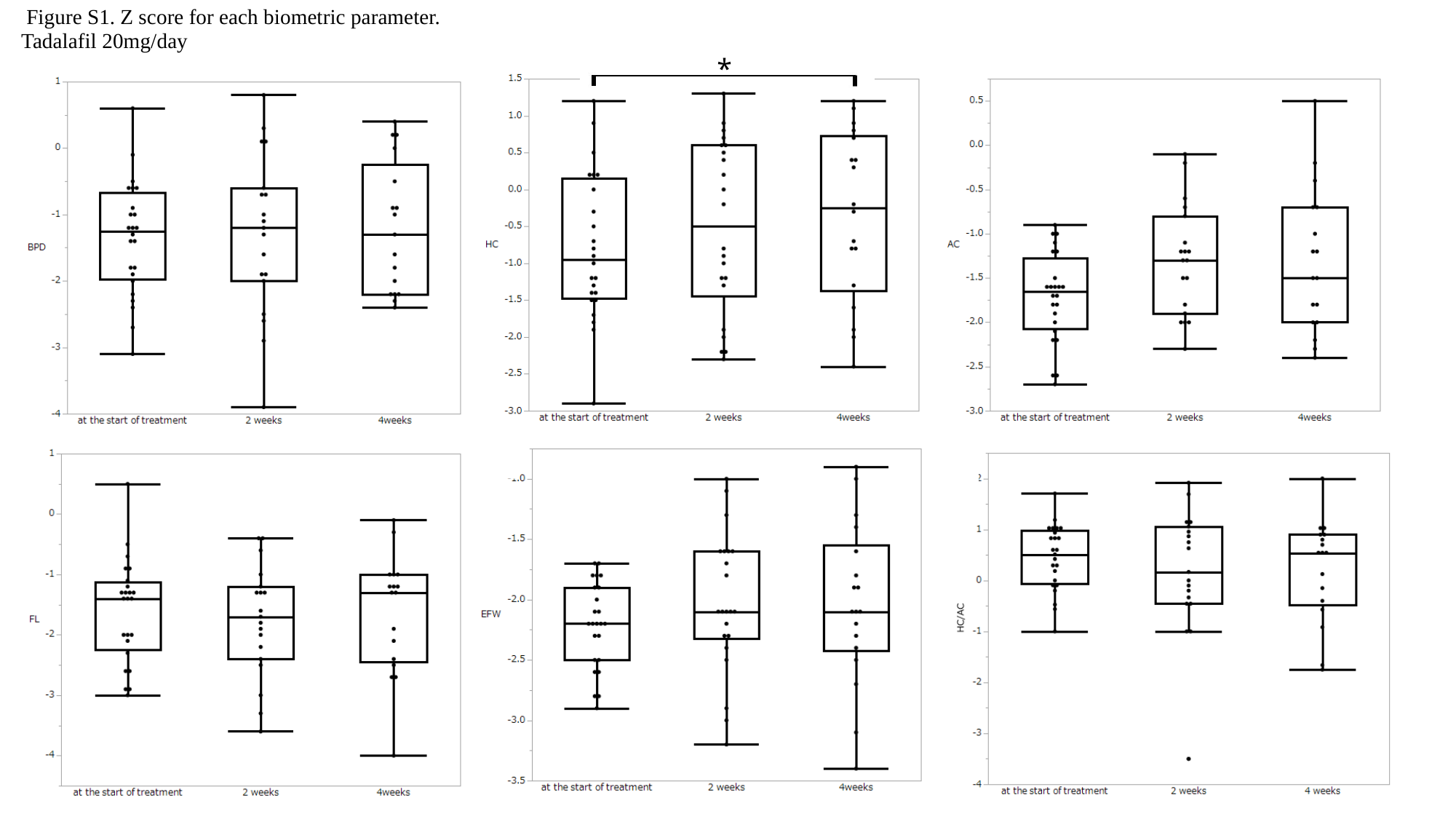

Figure S1. Z score for each biometric parameter.
Tadalafil 20mg/day
*

## Slide 2
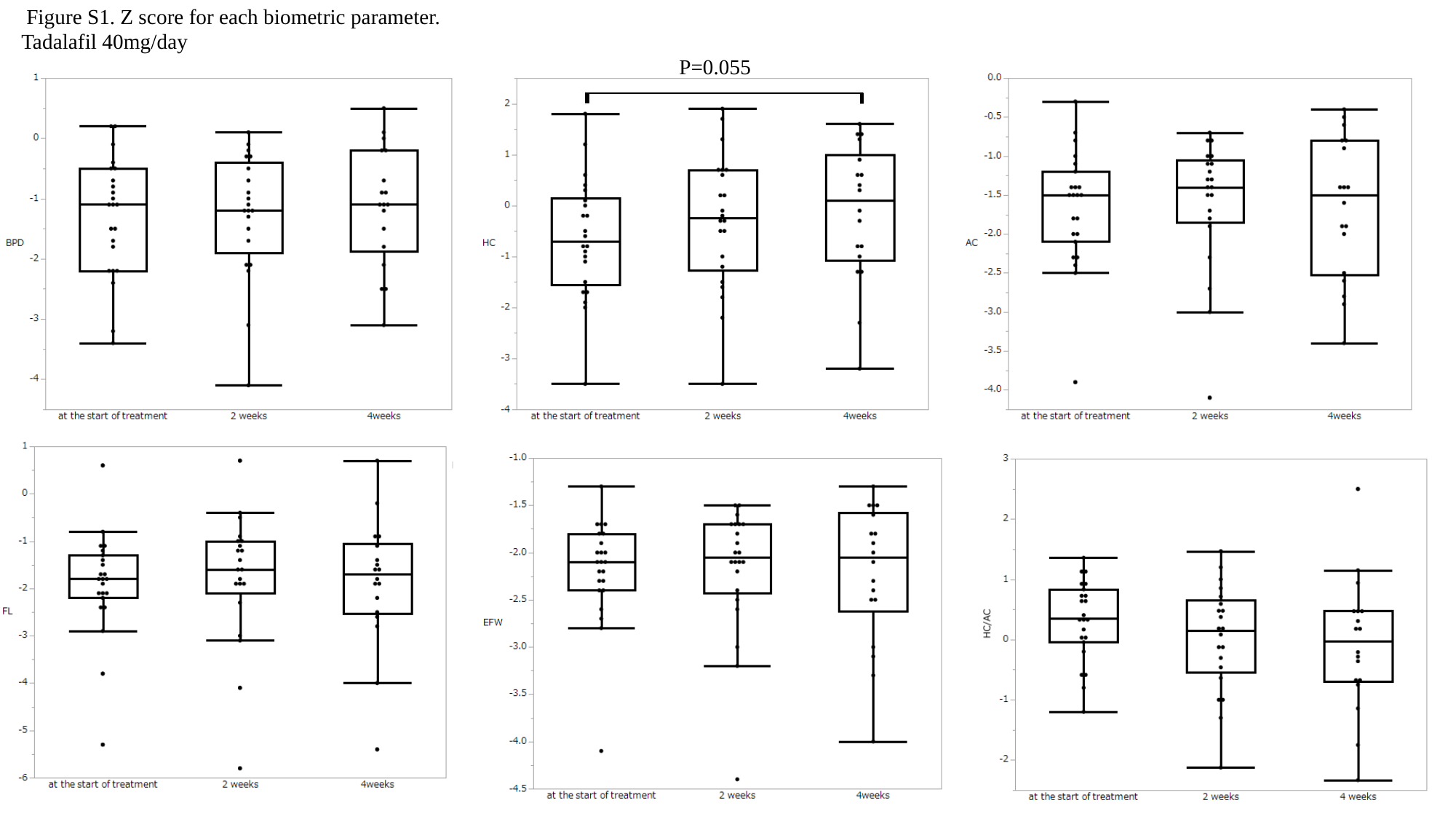

Figure S1. Z score for each biometric parameter.
Tadalafil 40mg/day
P=0.055

## Slide 3
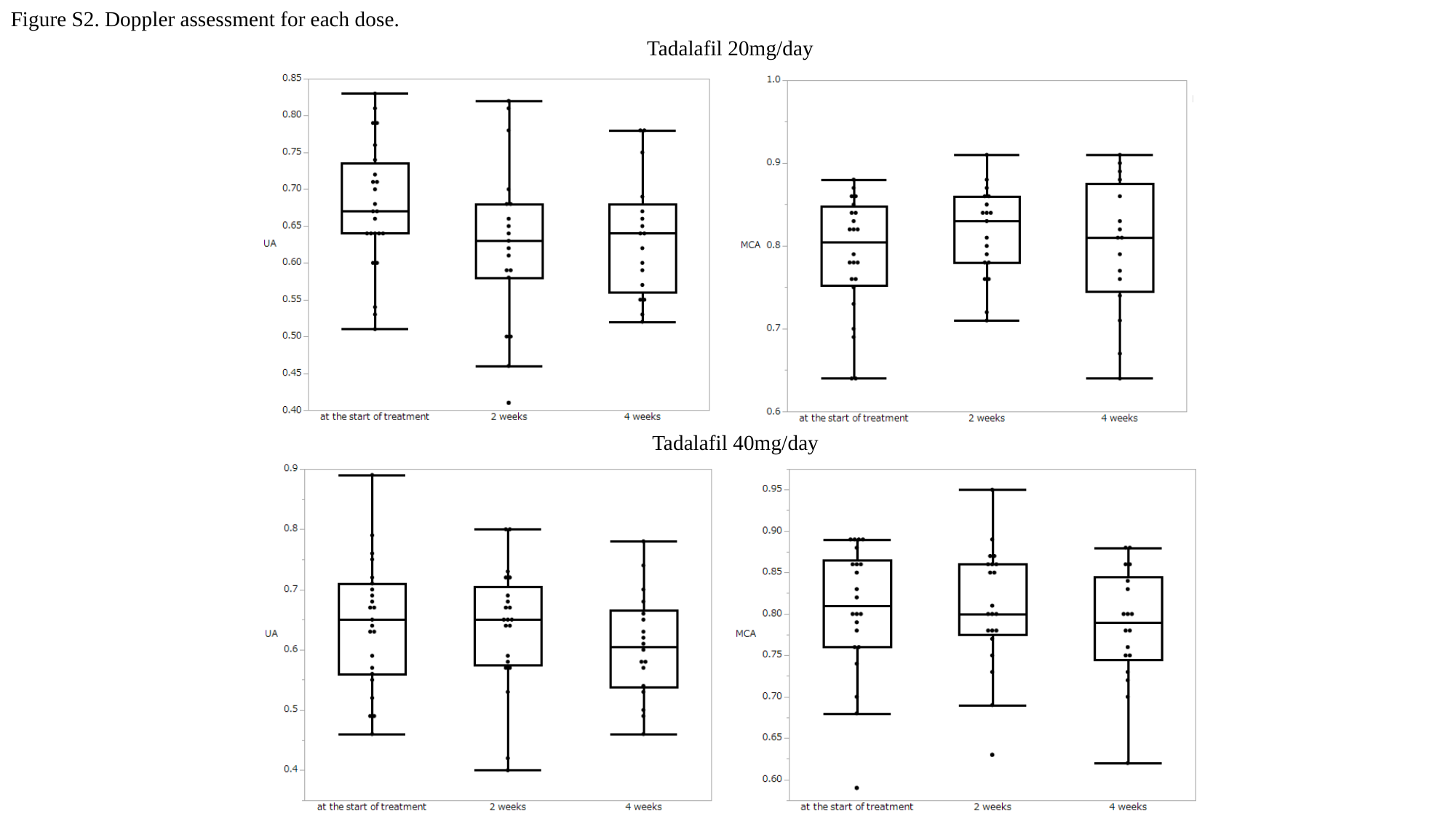

Figure S2. Doppler assessment for each dose.
Tadalafil 20mg/day
Tadalafil 40mg/day
