# Supplementary material for: Fetal Biometric Assessment and Infant Developmental Prognosis of the Tadalafil Treatment for Fetal Growth Restriction
Source: Medicina (Kaunas). 2023 May 8;59(5):900. doi: 10.3390/medicina59050900 (PMC10223650; doi:10.3390/medicina59050900)
Supplement: Supplementary file 1 [file medicina-59-00900-s001.zip › Supplemental_Appendix_Table.pdf]

Supplemental appendix

Table S1. Comparison of biometric parameter for each dose of tadalafil (10 mg vs. 20 mg vs. 40 mg)

		Dose of tadalafil			
		10 mg (n=3)	20 mg (n=17)	40 mg (n=18)	p value
BPD	2 weeks of treatment	-1.5 (-2.0 to -0.3)	-1.2 (-2.0 to -0.6)	-1.2 (-1.9 to -0.4)	0.98
	4 weeks of treatment	-1.4 (-1.9 to -0.9)	-1.3 (-2.2 to -0.3)	-1.1 (-1.9 to -0.2)	0.95
HC	2 weeks of treatment	-1.0 (-1.2 to -0.1)	-0.5 (-1.5 to 0.6)	-0.3 (-1.3 to 0.7)	0.72
	4 weeks of treatment	-0.4 (-1.3 to 0.6)	-0.3 (-1.4 to 0.7)	0.1 (-1.1 to 1.0)	0.80
AC	2 weeks of treatment	-1.2 (-1.3 to -1.1)	-1.3 (-1.9 to -0.8)	-1.4 (-1.9 to -1.1)	0.52
	4 weeks of treatment	-1.2 (-1.4 to -0.9)	-1.5 (-2.0 to -0.7)	-1.5 (-2.5 to -0.8)	0.39
FL	2 weeks of treatment	-1.5 (-2.2 to -0.1)	-1.7 (-2.4 to -1.2)	-1.6 (-2.1 to -1.0)	0.63
	4 weeks of treatment	-1.9 (-2.4 to -1.4)	-1.3 (-2.5 to -1.0)	-1.7 (-2.5 to -1.1)	0.59
EFW	2 weeks of treatment	-1.9*	-2.1 (-2.3 to -1.6)	-2.1 (-2.4 to -1.7)	0.80
	4 weeks of treatment	-2.1 (-2.1 to -2.0)	-2.1 (-2.4 to -1.6)	-2.1 (-2.6 to -1.6)	0.91

Data are shown as median DQ (IQR)
Analyses were done by Kruskal-Wallis H test.
*Values were the same for all 3 cases.

Table S2. Neonatal characteristics.

	Tadalafil treatment group (n=50)	Control group (n=10)	p value*
Birth weight (g)	1986 (1425-2188)	1929 (1435-2381)	0.94
Z score	-2.1 (-2.7- -1.6)	-2.2 (-2.4- -1.3)	0.66
Height (cm)	42.4 (40.0-44.9)	41.5 (39.8-44.4)	0.92
Z score	-2.0 (-2.4- -1.4)	-1.8 (-2.7- -1.4)	0.82
Head circumference (cm)	30.4 (28.6-32.0)	30.2(28.6-31.9)	0.98
Z score	-1.1 (-1.6- -0.6)	-1.0 (-1.3- -0.3)	0.46
Umbilical cord pH	7.28 (7.25-7.30)	7.28 (7.26-7.30)	0.92
BE (Base excess)	-3.6 (-5.7- -2.1)	-3.5 (-4.4- -2.3)	0.72

Data are reported as median (interquartile range), n (%)
* Wilcoxon rank sum test
